# Tool and techniques study to plant microbiome current understanding and future needs: an overview

**DOI:** 10.1080/19420889.2022.2082736

**Published:** 2022-08-10

**Authors:** Enespa, Prem Chandra

**Affiliations:** a Department of Plant Pathology, School of Agriculture, SMPDC, University of Lucknow, Lucknow, India; b Department of Environmental Microbiology, Babasaheb Bhimrao Ambedkar (A Central) University, Lucknow, India

**Keywords:** Plant microbiome, phytohormones, transmission electron microscope (TEM), polymerase chain reaction (PCR), DNA fingerprinting

## Abstract

Microorganisms are present in the universe and they play role in beneficial and harmful to human life, society, and environments. Plant microbiome is a broad term in which microbes are present in the rhizo, phyllo, or endophytic region and play several beneficial and harmful roles with the plant. To know of these microorganisms, it is essential to be able to isolate purification and identify them quickly under laboratory conditions. So, to improve the microbial study, several tools and techniques such as microscopy, rRNA, or rDNA sequencing, fingerprinting, probing, clone libraries, chips, and metagenomics have been developed. The major benefits of these techniques are the identification of microbial community through direct analysis as well as it can apply *in situ*. Without tools and techniques, we cannot understand the roles of microbiomes. This review explains the tools and their roles in the understanding of microbiomes and their ecological diversity in environments.

## Introduction

Microorganisms have a variety of roles in both human and environmental life. Despite their small size, these organisms have a significant impact on society, either positively or negatively [1]. Small organisms, on the other hand, can be found in almost any environment, including water, soil, and air, making microorganisms pervasive [2]. Microbe research is difficult due to our lack of understanding of them and their environments. Because microorganisms occur in large quantities in the natural environment, conventionally based methods do not provide enough data to fully comprehend microbes and their impact on the ecosystem, including plants [3]. However, due to a lack of relevant and up-to-date instruments and methodologies for understanding these microbes, numerous microbial species have remained unknown till today [4,5].

Different techniques/procedures are available for characterization and identification of microbes, such as isolation, purification of visible colonies, and microbe rearing, however, all methods are applied at the laboratory desk, and these methods are old [6,7]. Some techniques and instruments, such as culture technique and microscopy, have been widely employed in the past, but they provide a limited picture of the microbial world [8,9]. Many bacteria seem the same under a microscope, and many won’t thrive outside of their natural habitats. Because of a lack of tools and methodologies, only around 1% of microbes have been discovered. This is because certain microbes are viable but non-culturable (VBNC) while others are found in extreme environments such as very high or low temperature, pH, pressure, salinity, and so on [10,11]. As a result, early scientists only investigated a small number of bacteria, and a certain region of microbial habitat refers to just those microbes that have been produced in a microbial laboratory. However, increased culture is required for a complete investigation of microorganisms, which can only be done in the lab [12,13]. For the study of microbial diversity, there are commonly three methodologies. 1) methods that are culture-specific 2) Techniques that are not culture-specific 3) methods based on molecular biology. Culture-dependent approaches lose the majority of microbial species, making it impossible to investigate microbial ecology [14,15], but culture-independent and molecular techniques allow us to research microbial ecology more easily [16]. For bacterial identification, PCR amplification of the universal 16S rRNA gene is often utilized. 16 sRNA sequencing offers data at the taxonomic level of bacterial species, and it is a commonly used technology [17,18]. The comparable rRNA gene known as 18 sRNA is used to research higher microorganisms such as fungus [19]. If 18 sRNA does not provide enough information on the fungi, we go on to the internally transcribed spacer (ITS). ITS gives complete data for fungus taxonomy identification [20,21]. Although neither technique can identify a new species, this is a significant drawback of both techniques because they both rely on primers. Although both procedures cannot identify new species, this is a significant limitation of both techniques because both techniques rely on primers that detect just the target species/organisms [22–24].

All realms of life, on the other hand, are populated in a complex environment. Prokaryotic grazers, such as protozoa, fungi, nematodes, and oomycetes, can be important plant symbionts or destructors, while others are prokaryotic grazers found in most soils around the world [25,26]. In agricultural soils, the archaea domain participates in metabolic activities such as ammonia production, oxidation, and methanogenesis. In the environment, bacteria in the last domain serve a variety of activities, including plant growth and development, pathogen defense, bioremediation, biodegradation, and other industrial sectors [27]. They also operate in the manufacturing and service industries, producing ethanol, enzymes, acids, fragrances, and medications, among other things. Bacteria interact with the host plant and other bacteria in the ecosystem, therefore capturing as much variation of a microbiome as possible is critical [28]. To accomplish so, international methodologies such as metagenomics, meta-transcriptomic, meta-proteomic, and metabolomics must be used, which allow for the simultaneous appraisal and judgment of indigenous microflora across all domains of life [29]. Metagenomics is the direct extraction and cloning of genetic material from samples to determine the genomic diversity of microbes (culturable and unculturable), whereas metaproteomics, metatranscriptomics, and metabolomics, respectively, provide a sketch of community-wide protein abundance, gene expression, and metabolic activities [30,31].

In this review, we will look at the know-how and methods that have been used in the isolation and characterization of bacteria and microbiomes on a morphological and molecular level. We’ll also learn how to use metagenomics to identify VBNC organisms. These methods can also be used to investigate microbiomes, or microbe populations, in various contexts [32].

## Plant microbial community

To address the importance of the soil-associated plant microbiome to plant trait expression and ecosystem functions, the aspects of the plant microbiome covered in this overview are limited to the rhizosphere, rhizoplane (epiphytes), and internal endosymbiosis (endophytes) of the belowground organs of the plant [33–35]. However, this ignores the rhizosphere microbiome’s effects on aboveground interactions, including herbivory, pollination, and seed predation, as well as pathogen attack from aboveground structures [36,37].

**Figure 1.**
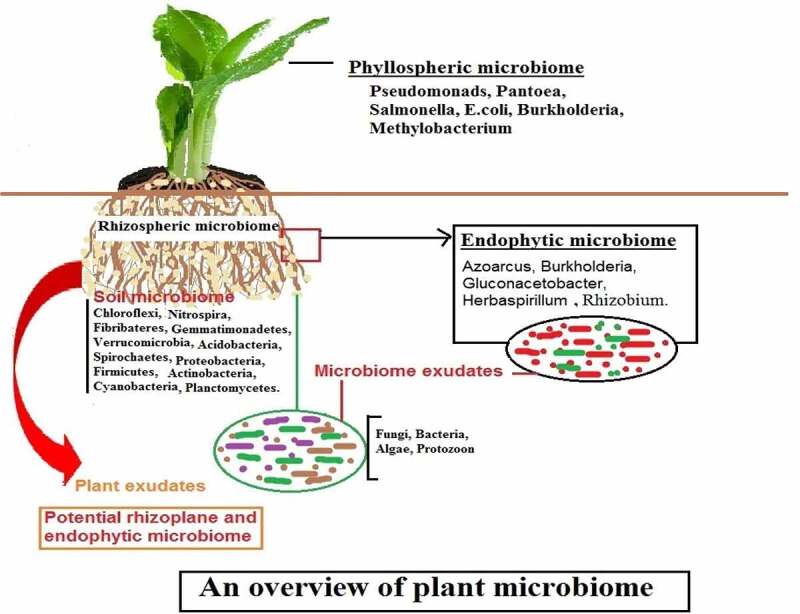
Role of the plant microbiome.

Plant-microbe interactions are important for plant growth and yield, and they have gotten a lot of attention recently [38]. This type of interaction is seen in all regions of plants, according to microbiologists and ecologists, but it is called a plant microbiome when it occurs in a specific portion of the plant [39]. “A plant microbiome is a specific place/region/habitats, such as roots, leaves, stems, and floral sections, where varied bacteria exist and display various interactions with the plants,” according to the definition [40]. These interactions may be mutual/beneficial/harmful given in Figure 1.

## Types of plant microbiome

Plant microbiomes are diverse, containing both beneficial and harmful rhizospheric microorganisms, as well as endophytic and pathogenic pathogens [41]. Plant microbiome is categorized into three kinds based on their occurrence and interactions with the plant: 1) Rhizospheric plant microbiome; 2) Phyllospheric plant microbiome; and 3) Endophytic plant microbiome.

### Rhizospheric plant microbiome

Soil has a variety of microorganisms, which divide into two zones based on their availability to the plant [42]. The rhizosphere is a zone characterized by increased microbial mass and soils that directly surround plant roots. The rhizosphere is further separated into two parts: a) edaphosphere (one side bordered by soil region) and b) histosphere (the other side surrounded by plant tissues), both of which play a vital role in rhizosphere development [43,44]. Rhizospheric soils have a high water-holding capacity, indicating that there are many mutual interactions between microorganisms and plant roots in the soil, as well as improved nutrient availability. Rhizoplane refers to soil particles that are closely adhered to the root surface [45,46].

Fungi, protozoa, archaea, nematodes, oomycetes, bacteria, algae, and viruses are all frequent creatures found in the rhizosphere, and these organisms are referred to as rhizo-microbiomes [37,47]. These creatures dwell in the rhizosphere and feed on the plant’s nutrients (organic acids, sugars, amino acids, fatty acids, vitamins, and growth hormones) [48]. Plant growth-promoting rhizobacteria (phosphate solubilizing bacteria, nitrogen-fixing bacteria, biological control bacteria), cyanobacteria, fungi, mycorrhiza, protozoa, and pathogenic bacteria, fungi, virus, and nematodes are among the organisms that have been well studied for their beneficial effects on plant growth and development [27]. Another type of bacteria to consider is *Pseudomonas aeruginosa*, which improves plant growth and production but can cause sickness in people [38,49]. Plant growth and fitness are influenced by rhizospheric microbiomes, which can be beneficial or harmful. It is positively influenced by phytohormone secretion (indole-3 acetic acid, gibberellin), production of siderophore and ammonia, solubilization of phosphate, zinc, and potassium, and indirectly by decomposition of detritus, nutrient cycling, pathogen inhibition, secretion of stress hormone (ACC deaminase), and stimulation of the plant immune response [50]. However, certain bacteria function as pathogens, reducing crop productivity [51].

### Phyllospheric plant microbiome/aerial plant surface microbiome

The phyllosphere is the world’s second-largest microbial habitat. By colonizing severe, stressful, and changing settings, the microbial community in this region performs a dynamic function [52,53]. It is a significant point of entry for phytopathogens into plant tissues, where they cause disease. Furthermore, they offer a unique location for easily comprehending the interaction between microbiota and plants, as well as the methods by which distinct microbial populations sustain their populations in nature [54,55]. In comparison to the rhizosphere or endophytic zone, the phyllosphere has a low nutritional content [56].

On the phyllosphere, microbial population colonization is different, but it is influenced by the leaf’s stomata, hairs, and veins [57]. On the leaf surfaces, 107 microorganisms per cm^2^ colonize [58]. The phyllosphere is a much more interesting place, where microbes live in the presence of large fluctuations in temperature, radiation, moisture, and light throughout the day and night. Plant metabolism changes as a result of these environmental factors and the phyllosphere microbiome are affected [59].

### Endophytic/ root interior plant microbiomes

Endophytes are microorganisms that dwell inside plant tissue, whereas the endosphere is the surrounding environment [60]. Endophytic bacteria are typically thought to be non-disease causing agents because they cause no obvious symptoms on plants [61], but researchers have recently uncovered several pathogens that are dependent on the host genotype and environmental factors.

Endophytes are thought to have started in the rhizosphere, although they exhibit unique characteristics from rhizospheric microbes, implying that not all rhizospheric microbes can penetrate the plant [62]. When bacteria penetrate a plant, they change their physiological/metabolic processes and adapt to the host’s inner environment [3].

## Tools and techniques to the understanding of plant microbiome

There are new methodologies and procedures available to assist in the research of microorganisms that reside in a biome, allowing for accurate microbial ecology values [63]. The abundance of species, population size, species consistency, and species distribution are all factors in microbial ecology [64]. Measurement of microbial diversity is routinely done using morphological, biochemical, and molecular approaches. Molecular-based approaches, for example, can provide taxonomy-level information [65].

Some molecular approaches, including G + C percentage, restriction fragment length polymorphism (RFLP), DNA hybridization, and community-level physiological profiles (CLPP), are useful for identifying aquatic populations without providing any data [66]. Many DNA fingerprinting techniques, such as denaturing gradient gel electrophoresis (DGGE), temperature gradient gel electrophoresis (TGGE), repetitive PCR, arbitrarily PCR, and terminal-restriction length polymorphism (T-RFLP), are now available and widely used in the identification of microbial species by retaining polymerase chain reaction from environmental samples [67,68]. Researchers, on the other hand, can’t utilize diversity metrics since microbiomes don’t have a specific diversity and fluctuate as the environment changes [69]. First and foremost, the origin of the community must be known to comprehend the secret of microbes and plants in a specific habitat [70]. Different tools and strategies are required to do this. Table 1 lists the most important instruments and strategies for learning about plant microbiomes.

**Table 1.** Major tools and techniques for studying of plant microbiome and their merits and demerits [96].

S. No.	Tools/techniques	Merits	Demerits	Major drawbacks	Explanations
1.	Microbial cultivation by plating methods	A simple approach enables a more in-depth analysis of colonies, such as species identification and metabolic features.	It’s tough to distinguish between morphological colonies because the resolution capability is smaller and there aren’t any images.	Only culturable bacteria are detectable.	It’s a crucial technique for molecular-based identification analysis.
2.	Electron microscopy-SEM-TEM	It’s a valuable approach for studying the surface of diverse microorganisms and determining the location of ecological niches, as well as learning about bacteria’ cytotoxic pathogenic capabilities.	These are huge pieces of equipment that need time-consuming and expensive sample preparation. It necessitates an isolated location with a steady voltage. It generates graphics in black and white.	The main disadvantage of this method is that the sample size must be restricted to withstand the high vacuum pressure, electron transmission, and fit inside the analyzer.	Images are created by the interaction of electrons with the material in these procedures.
3.	Nucleic acid extraction/DNA sequencing	For searching genes in the soil microbial community that produces reliable and quick results. The scientific community prefers this strategy the most.	Incomplete and partial sampling is common. It is a more costly method that necessitates a lower throughput and post-PCR analysis.	The consistency of the chemical and the purity of the sample may limit the scope of the investigation.	It is based on a molecular approach for analyzing nucleic acid extraction biases, which must be reduced.
4.	PCR/qPCR	PCR is a procedure that is quick, simple to use, sensitive, repeatable, and culturally independent. The method is low-cost, time-saving, requires little input, and has a high throughput. It is common to practice using routine procedures to detect and quantify microorganisms.	It necessitates a high level of skill and technical support for settling, as well as expensive equipment costs, the use of primer dimers, and non-specific amplification.	Microbes in the range of 0.1 to 1% can be detected.	It is a common method for detecting microbial species from soil or leaf samples using molecular techniques.
5.	Molecular finger printings techniques (T-RFLP, DGGE, TGGE, SSCP, RISA, LH-PCR).	It can easily compare samples, increasing the odds of discovering various fingerprints from the same sample.	A maximum of 1000 members of the target community are contacted.	The nature of separation processes is a key flaw in molecular fingerprints, and interpretations must be done with caution.	Used to collect data on the microbial population through comparison research, has evolved into routing fingerprinting.
6.	RAPD	It’s a really simple, low-cost, and time-consuming procedure. Any DNA sample can be used with RAPD. This method does not necessitate the use of target genomic data.	High molecular weight and pure genomic templates are required.	The fundamental disadvantage of this procedure is its lack of repeatability.	RAPD is a form of PCR reaction in which small portions of primers are duplicated in random order.
7.	Clone genomic libraries	It’s a simple way to look into any gene in a community or at the diversity level.	Sample preparation is a time-consuming process. Because of its huge genome, it does not produce adequate results in eukaryotic creatures.	The most significant disadvantage of this method is identifying a clone from the library that scrambles a certain gene or gene of interest.	It is a really good approach, however, it does not give a fair impression of the targeted gene.
8.	Stable isotope probing	It has a positive impact on the dynamic community. It establishes a link between community structure and function.	Difficulties of opportunistsConcealing the information.	Microorganism activity is quite low, which makes probing difficult.	It is a procedure that is used in situ and is widely acknowledged in the scientific community.
9.	Microarrays	It is highly sensitive, low-cost, and provides direct information on species sequencing. It has an extremely high throughput. It does not necessitate the use of any special equipment.	It only produces chipped genes, is more time consuming, and expensive. When closely related members of the same gene family are tested, the results are negative.	Cross hybridizations with minimally homologous sequences are the most significant disadvantage.	It allows for high-throughput analysis across multiple locations.
10.	Next generation technique (NGS)–metagenomics-transcriptomic	It is an effective method for conducting comparative investigations. It has the ability to examine everything at once and with a high throughput.	It is more costly and time demanding. In NGS, method error is a big issue.	Researchers make poor interpretations due to flaws (method/technical procedure).	It can be utilized in a variety of investigations, but the data must be interpreted carefully.

### Microscopy

Researchers employ a variety of microscopes to study the plant microbiome, including light, compound, dark field, bright field, confocal, and fluorescence microscopes [71]. However, because these microscopes have a smaller focusing point, they are not suitable for in-depth research. Electron microscopes, such as the scanning electron microscopy (SEM) and transmission electron microscope (TEM), can overcome these restrictions since they have a high-resolution power attached to the usage of an electron beam, as opposed to light or compound microscopes [72]. Microorganisms and plants on the surface and inside the cell can be spotted using an electron microscope [73]. Furthermore, the microbiome’s habitat, niche, host, and behavior can all be explored [74]. The microscope aids in the colonization of microorganisms on or inside the plant surface, as well as the understanding of their role in the plants [75]. Because it is a basic requirement of microorganisms for sustenance, and because of the nature of the habitat, effective colonization is a vital point in plant-microbe interactions [3]. Thus, plant colonization has long been an important issue in studies on the routes and roles of these organisms with plants [76], and microscopy aids in the viewing of microorganisms in their natural habitat and relationship with plants [77]. Furthermore, this method enables the tracking of microorganisms [78].

### Nucleic acid extraction

Understanding the plant microbiome requires the use of nucleic acid extraction, which is one of the most significant technologies in biology. A nucleic acid extraction is an old approach that has been well refined in the twenty-first century [79]. Nucleic acids are extracted in three processes, depending on the sample and downstream application: a) breaking the samples (tissue or cells); b) removing lipids, proteins, and other contaminants from the nucleic acid; and c) transferring the nucleic acid to a buffer solution for storage [80]. However, numerous commercial molecular kits for nucleic acid extraction are currently available on the market. However, the identification of DNA and RNA can be done using both traditional and kit approaches, and both methods are used to extract quantitative and qualitative nucleic acid analyses [81]. Furthermore, it is expected that the same standardized procedure is employed, as each method will have its preferences in terms of nucleic acid quality and amount [82,83].

### Nucleic acid hybridization

In the study of the plant microbiome, nucleic acid hybridization (DNA-DNA and RNA-DNA) is a useful technique [84]. Hybridization reaction occurs when two harmonizing single-stranded nucleic acids form a partial or entire double-stranded nucleic acid by a specific-sequence interface [85]. Nucleic acid is analyzed quantitatively and quantitatively utilizing specific probes in this approach [86]. The probe, which is recognized sequences spanning in specificity from domain to species and is identified with markers at the fifty end position, is often taken [87]. There are a variety of nucleic acid hybridization procedures available, with FISH (fluorescent in situ hybridization) being one of the most popular [88].

This method can be used to investigate the spatial dispersion of a microbial population in a different place [89]. However, due to a lack of sensitivity and the presence of high copy numbers in comparison to dominant species in a sample, we cannot directly extract nucleic acid from environmental materials using this method [90]. As a result, analytical tools can be used to overcome these constraints, and PCR is a good option. Membrane hybridization is another prominent technique utilized by researchers in addition to FISH [91]. Denatured RNA or DNA is immobilized on inner support in such a way that self-annealing is prevented while the residual sequence (bound) is present for hybridization with tagged probes in the membrane method (single or double-stranded). Furthermore, the membrane is extensively washed to remove the unattached probe, and a low case of matched hybrids follows the nucleic acid hybridization reaction [92].

### Polymerase chain reaction

One of the most essential techniques for increasing or amplifying a certain target sequence of DNA is the polymerase chain reaction (PCR) [93]. In PCR, the targeted sequences are chosen from the nucleic acid sequence, such as repeated sequences or specific genes [94]. The PCR process takes at least 35–40 cycles to complete and is divided into three parts based on temperature: One cycle consists of 1) denaturation, 2) annealing, and 3) elongation. 16S rRNA gene primers are known as universal or species-strain primers [95].

It’s a promising PCR amplification for bacterial species identification and phylogenetic reasons [96]. The PCR employs nonspecific dyes (SYBER Green I and SYBER Gold) that bind to double-stranded DNA [97]. PCR develops numerous variants based on the type of sample to justify the isolation and quantification of live bacterial numbers at the same time. Multiplex PCR is a fantastic example for separating mixed bacterial pathogens from a sample as well as differentiating multiple species belonging to the same genus [65,98].

The earliest PCR, on the other hand, is unable to detect live or dead bacterial cells. However, this problem can now be rectified using a cutting-edge technology known as reverse transcriptase PCR (RT-PCR) [99]. The reverse transcriptase enzyme powers RT-PCR. This technology, on the other hand, is sensitive yet does not require pre-enrichment operations, and it also takes less time than traditional PCR. Furthermore, VNC cells cannot be cultivated using standard PCR or simple laboratory methods, but we can detect them using RT-PCR [100,101].

Exonuclease activity generates a luminous and detectable signal in RT-PCR. A signal is created with each PCR cycle. The generated signal enables real-time detection through real-time PCR. When a signal permits a specific threshold level, the signal is transformed into forecast target gene numbers based on a pre-established calibration line with ordinary target DNA [102]. RT-PCR also assesses the magnitude of gene effects in local settings as well as the degree of gene expression in microhabitats. As a result, it may be possible to more precisely map microflora and their utility to the soil, plants, and other places using this technique [103].

### DNA fingerprinting

Because every organism has unique DNA, DNA fingerprinting is a unique approach for identifying an organism based on DNA properties. It is primarily utilized in forensic sciences, although it is currently used in a variety of fields, including the study of plant-microbe interactions [104]. In the fingerprinting techniques, restriction fragment length polymorphism (RFLP), polymerase chain reaction-restriction fragment length (PCR-RFLP) [105], denaturing gradient gel electrophoresis (DGGE), terminal restriction fragment length polymorphism (T-RFLP), temperature gradient gel electrophoresis (TGGE), single-strand conformational polymorphisms (SSCP), ribosomal internal spacer analysis (RISA), length heterogeneity-PCR (LH-PCR) are involved [106,107].

These strategies aid in the study and comparison of data from microbial communities in a sample. However, some PCR-based approaches for microbial community characterization were outmoded, but many new post-PCR analytical techniques have emerged in recent years [108]. The main advantages of this method are that it allows for a comparison of the microbiome’s morphology, composition, and diversity in samples such as soils. Another advantage of this technique is that it can distinguish between viable and nonviable cells in the microbial population, which is something that no other fingerprint technology can do [109].

### Denaturing gradient gel electrophoresis

DGGE is a very useful technology for detecting the microbial population directly from environmental materials, which means it does not require microbe rearing. PCR-DGGE, phylogenetic DGGE, and functional gene DGGE are the three most common forms. Only a few samples are required for microbial community characterization in PCR-DGGE [110]. This method detects specific clusters of microorganisms from various plant zones, such as roots and leaves.

On polyacrylamide gels with denaturing gradients, the principle of PCR-DGGE is carried out. However, while this technique was originally developed for mutation analysis, it is currently employed in a wide range of applications, such as detecting microbial communities in environmental samples [111]. This technique has the advantage of simultaneously observing many samples and assessing microbial communities based on ecological and historical differences [112,113].

Phylogenetic study of bacteria using 16 sRNA genes is commonly employed in PCR-DGGE currently. This is not a new notion, as similar techniques have been in use since 1990 [114]. The single species of the community can be identified using phylogenetic DGGE [115,116] by removing DGGE bands from the gel and sequencing them, or by creating clone libraries of 16S rRNA that are separated using DGGE [115]. Muyzer *et al*. [117] reported that the 16S rRNA gene is employed as a molecular biomarker for microbial species in a population. The most important thing to remember when using partial 16S rRNA gene sequencing is to be cautious when interpreting the results. V4-V5 are appropriate sections for phylogenetic analysis when compared to full-length sequence data [118]. However, the DGGE technique has some limitations, such as microbial DNA extraction and community analysis from environmental samples. Another limitation is that while one strain produces only one band, in some species two or more bands have been observed, and thus we cannot estimate the true microbial diversity data [110].

The data of soil health, quality, and function is provided by the functional gene-based DGGE. We may simply link reduced soil microbial diversity to poor soil functioning this way [119]. The study of coding proteins genes entangled in critical biome practices has gotten a lot of interest in the past few years [120]. Furthermore, functions in which the genes are accommodated by one or more species of bacteria were postulated. In comparison to extremely unnecessary bacteria species/groups, interruption inducing bacteria species/groups affect the functioning of soil [121].

Furthermore, functional genes for nitrogen fixation, denitrification, and other processes are abundant in bacterial species. Since the previous few decades, there has been a surge in interest in gene databases, including primer design, gene function, and gene identification [122]. The gene producing nitrate reductase narG, nitrite reductases nirS and nirK, encoding dinitrogenase reductases nifH, and amoA encoding the ammonia monooxygenase have all been used as substitutions to track changes in soil functional gene diversity [123,124]. The nifH has recently been utilized to investigate the influence of GM white spruce on soil N_2_ fixation communities. However, the authors failed to mention the GM plant’s significant impact on N_2_ fixation ecosystems [125]. Another study used the PCR-DGGE approach to positively trace the phlD gene, which codes for diacetyl phloroglucinol (DAPG), an antagonistic chemical generated by pseudomonads [126].

### Clone libraries

A clone library is a collection of DNA fragments that have been cloned into vectors and have been used by researchers to identify and extract those DNA fragments that they are interested in studying further [127]. cDNA libraries and genomic DNA libraries are the two types of libraries that exist. The cDNA library is made up of clones and contains reverse-transcribed mRNA, but it lacks DNA sequences corresponding to genomic areas that aren’t expressed, such as 5’ and 3’ noncoding regions, and introns. Genomic libraries, on the other hand, contain huge amounts of DNA in the form of bacterial, bacteriophage, or other synthetic chromosomes [128].

Clone libraries provide immediate access to information on the microbiome’s targeted gene sequences. This method involves joining PCR-generated replicons to a suitable vector plasmid [129]. In addition, using the transformation procedure, the synthesized DNA is cloned into an appropriate host such as *Escherichia coli*. Following transformation, plasmid extraction can be used to remove cloned replicons from the inserted vector, which can then be sequenced and studied using databases [130]. Chimeras (a single bacterial cell with two different genotypes) are generally eliminated during this procedure.

Because the strain’s sequences are evaluated separately, this technique is far superior to phylogenetic analysis or fingerprinting techniques [131]. The capacity to directly obtain and evaluate novel strains is a major benefit of this technology, which improves our understanding of the soil microbial population [132]. However, other researchers claim that clone libraries are a time-consuming method because many techniques have progressed in recent years, allowing for a more comprehensive understanding of bacteria and functional genes in a microbiome [109].

### DNA microarrays

For studying the soil microbiome, DNA microarray is an excellent technique. The term “DNA chip” or “biochip” is also used to describe it. This technique is used to examine the genetic constitutions of several sections of an organism’s genome or to evaluate gene expression levels at a large number of genes at the same time [133,134]. The sample’s DNA is extracted and amplified using PCR in this procedure. A microarray is used to analyze additional DNA samples that have been tagged. A thick range of oligonucleotide probes is inserted on the microarray, ranging from 10 to 1000 [135]. Probes could be 16S rRNA gene fragments or functional gene fragments. The DNA samples must be homogeneous to the probes on the chip for them to bind or hybridize. The chip’s signals are digitally analyzed after binding [136]. Chip contributes to our understanding of phylogenetic diversity, functional genes, and community composition in this way. When the material is extremely complex, such as soil, however, analysis can be difficult [137].

Cross-hybridization, on the other hand, is a serious concern with microarrays. At least 11 or more short oligonucleotides have been designed to overcome the challenge, allowing dissimilar matches to be distinguished from similar perfect matches [138,139]. According to the number of probes and design [140], there are two types of microarrays: a) geochips and b) phyloarrays. Over 24, 000 probes cover over 10,000 genes scattered among more than 150 functional categories enmeshed in carbon, sulfur, phosphorous, and nitrogen cycling on the geochips [141,142]. They’re utilized to collect soil samples. Geochips are utilized on Antarctic soils to analyze various cycles such as carbon, nitrogen, and other elements. Microarray binding holds a lot of promise, including the possibility of creating a “universal microarray.” It explains the state and condition of the soil [143].

### Next generation OR omics tools and techniques

Metagenomics is the most recent technology to be developed. It’s a cutting-edge technique. Metagenomics is the study of bacteria’ whole genomes retrieved from environmental materials. Metagenome gives a demonstrated understanding of species composition, genetic diversity, inter-species interactions, and species evolution in the context of typical civilizations’ environments [30]. Several sophisticated omics sequencing techniques, like pyrosequencing and Illumina sequencing, were established before the last several decades. These methods are useful in metagenomics and metatranscriptomics research.

For example, soil samples were identified using 454-based pyrosequencing and DNA or RNA extraction [144]. Multi parallel sequencing by synthesis is used in this technique, with luciferase enzyme detection and pyrophosphate release detection of the produced light [145]. Both Illumina and pyrosequencing, on the other hand, go through three steps of traditional sequencing: template preparation, library preparation, and actual capillary sequencing [146]. Due to multi parallelity, the sequencer can generate anything from 100 to millions of 450 base pair (bp) readings in a single run [147].

The sensitivity of next-generation sequencing, when collected straight from a soil sample, is investigated for its competence and impartiality [148]. As a result, the depiction and conclusion bias of the results are determined by the extraction of DNA from the soil sample [149]. Although the 454 sequencers are used directly for the development of base reads because it produces longer reads, the Illumina sequencer may be utilized to fill in gaps in the 454 generated sequence data because of its high throughput [150,151].

### Metabolomics

Metabolomics research aims to learn more about a biological system’s small molecule metabolites under specific conditions. Primary and secondary metabolites make up the metabolome in general. Plant defence mechanisms have a large diversity of secondary metabolites compared to other complex biological systems [152–154]. Herbivores and microorganisms find the majority of them poisonous or repulsive. Metabolomic compound analysis yields metabolic profiles and fingerprints, as well as the identification of novel biomarkers, which can be combined into microbiome research for a more holistic understanding of the plant microbiome [155].

### Analytical technologies used in metabolomics

Nuclear magnetic resonance (NMR), gas chromatography-mass spectrometry (GC-MS), and liquid chromatography-mass spectrometry (LC-MS) are the most common technologies utilised in metabolomics (LC-MS). MS-based approaches are substantially more sensitive than NMR at detecting metabolites [156]. MS samples, on the other hand, necessitate extensive preparation, and detection is limited to metabolites that can ionise into the detectable mass range. For chemicals that are difficult to ionise or dissolve, or that require derivatization for MS, NMR has certain advantages [157].

Targeted and untargeted techniques to metabolomics have hitherto been separated, however this may change in the future [158]. Pre-processing, annotation, post-processing, and statistical analysis are all steps in the analysis of data generated using these technologies (NMR and MS). These procedures are generally customised to the analytical technology [159]. To adjust discrepancies in peak shape width and location caused by noise, sample differences, or instrument parameters, pre-processing procedures are used. There is currently no gold standard pipeline for data pre-processing [160].

A metabolite must be compared to at least two orthogonal properties of an authentic chemical standard evaluated in the same laboratory using the same analytical methodologies as experimental data, according to the Metabolites Standard Initiative (MSI) [161]. Because most metabolites do not have chemical criteria, they cannot be completely identified. As a result, MS annotation tools are classified into several levels of annotation. Metabolites can be detected using NMR by simply comparing data from internet databases [162]. This restricts the results to the content of the relevant databases [163].

### Metatranscriptomics

Metatranscriptomics is a method for identifying active genes or pathways in a microbial community by sequencing the total message RNA (mRNA). This procedure entails extracting total RNA from microbial communities, eliminating ribosomal RNA (rRNA) to obtain high amounts of mRNA transcripts, reverse transcribing mRNA into cDNAs, ligating adapters, and sequencing with NGS [164]. Metagenomics and metatranscriptomics are frequently used jointly to give assembled genomes as templates for transcript mapping. Top Hat and HISAT, as well as Cufflinks and HTSeq, have been developed for this purpose. Metatranscriptomics has been successfully applied to a wide range of settings, including soil, sediment, gut microbiomes, and activated sludge. It’s a useful method for deducing community function and activity, as well as identifying novel pathways in uncultured microorganisms [165].

### Proteomics

The study of proteins in a microbial community extracted from an environmental sample is known as metaproteomics. Metaproteomics, unlike other -omics techniques, gives direct evidence for proteins, post-translational modifications, protein-protein interactions, and protein turnover, all of which represent the structure, dynamics, and metabolic activities of microbial communities [166]. Metaproteomics mostly use mass spectrometry-based proteomics technologies [167,168]. Metaproteomics has been used in plant microbiome studies to assess bacterial communities in the phyllospheres of tree species in a pristine Atlantic Forest [169], to investigate the response of the plant PGPB Bacillus amyloliquefaciens FZB42 to the presence of plant root exudates [170], and to determine the differences in soil protein abundance in plant sugarcane and rat Despite its success, metaproteomics in the plant microbiome is limited due to reduced protein expression in plant microbial samples and limited database information [171].

Metaproteomics is a technique for assessing microbial activity in an ecosystem at a certain moment using protein expression [172]. Metaproteomics, unlike metagenomics and metatranscriptomics, uses liquid chromatography tandem mass spectrometry (LC-MS/MS). The procedure begins with protein extraction, followed by LC-MS/MS 1generation of MS spectra, and finally, comparison of spectra with peptides from thousands of proteins from various taxonomic groups [173]. These comparisons can be made in two ways: by exploring current protein/peptide databases or by comparing to theoretical peptide spectra created in silico from metagenomes from the same sample or from similar environments [174,175]. Metaproteomics is a potent technique for deciphering active metabolic processes in various contexts in a more direct manner than metagenomics or metatranscriptomics. This method has been used to study soils [176], sediments, marine habitats, freshwater systems [177].

### Biosensor

Environmental samples such as air and seawater can be monitored for hazardous compounds using biosensors [178]. Nucleic acid amplification techniques, mass spectrometry methods, and receptor-ligand binding assays are the three most used approaches for multiplex detection from complicated matrices. By improving signal transduction, nanotechnology has been used in biosensors to improve sensitivity and performance of assays [179]. Nanotechnology is the process of creating structures, gadgets, and systems that have unique properties that can be controlled by changing the size and form of materials at the nanoscale scale [180].

## Advantages

1. To utilize these instruments to arrest and study the accumulative multifaceted data and information, new ways and approaches for evidence progress are required to build new methods and approaches [181].
2. Tools and procedures improve the research of microbial communities and evidence of these communities [113].
3. It aids in time management and data organization.
4. Some approaches, such as metagenomics, allow us to glimpse into the hidden microbial world, which aids in the study of viable but nonculturable (VBNC) bacteria [30].
5. Tools and techniques can be used to understand the connection between microbiomes and their hosts, as well as the link between symbiotic, mutualistic, and commensalism variety and functions [182].
6. Modern omics approaches to aid in the development of novel tactics and the conduct of research to obtain a detailed report on the microbiome and its expression, as well as the level of plant genome expression monitoring [183].
7. The finding of numerous new sequences, the ultra-high throughput of sequences, and the lack of preferences are all advantages of metagenomics [184].
8. Although the huge sequence of data obtained will necessitate the use of sophisticated bioinformatics software for processing, metagenomics does not [185].
9. Tools and procedures are also useful in the study of microbial metabolites and their interactions with plants [186].
10. The use of approaches can be seen in changes in diversity as a result of specific treatments, as well as in management strategies for soil diversity and production [187].

## Disadvantages

1. When working with a complex sample, such as soil, tools can cause issues.
2. One of the biggest drawbacks of this method is the high cost of the instrument used in microbiome research [32].
3. Until now, important tools that will be employed by the majority of researchers are unknown, implying that a researcher’s lack of skills is a big difficulty in comprehending the plant microbiome [188].
4. For a microbiome researcher, data interpretation, such as metagenomics, is a big difficulty [189].
5. An omics technology like pyrosequencing or Illumina is a lengthy procedure [190].

## Concluding remarks

We provide an overview of almost traditional molecular methods for accessing the soil microbiome in this review paper, including microscopy, nucleic acid extraction and hybridization methods, fingerprinting, PCR, and PCR-based techniques, as well as information on the development of a novel method and its application to environmental samples. Microarrays and metagenomics, for example, are new approaches that aid in the research of microbiomes that are visible and hidden in the world. The information on microbiome communities is derived from omics methods’ data. Furthermore, the roles of microorganisms in a given community may be understood. Metagenomics and metatranscriptomics aid in the in-depth investigation, and this is an interactive method that occurs in this microbial habitat. As a result of the application of molecular tools and methodologies, we have made significant progress in our knowledge of microbial communities in microbiomes. The nature of the sample, the collection, and the habitat are all important aspects of molecular identification. However, given the overall study of these techniques used to study variegated microbial communities of soil and the analytical power suggested by devouring culture microorganisms, it is strongly recommended to use a polyphasic analytical method to analyze soil and other environmental samples in these types of studies. The authors also mentioned the plant microbiome and how many different types of microbiomes exist in nature.

In addition, the positive and negative roles of microorganisms are reviewed in this review work. When studying the microbial ecology of natural habitats, ancient culture-based techniques are overpowering, but they are exceedingly unfair when studying microbial samples. Microbial ecology studies employing culture nondependent molecular procedures have ushered in a new era of microbial variety, thanks to recent advancements in the omics era and sequencing technologies.
